# Determining the Risk of Spontaneous Bacterial Peritonitis due to increase use of Proton Pump Inhibitors among cirrhotic patients with ascites

**DOI:** 10.12669/pjms.37.4.3476

**Published:** 2021

**Authors:** Bashir Ahmed Shaikh, Zahid Ali Shaikh, Aftab Hussain Shah, Aneel Kumar

**Affiliations:** 1 Bashir Ahmed Shaikh, FCPS, FRCP. Chandka Medical College Hospital, Shaheed Mohtarma Benazir Bhutto Medical University (SMBBMU), Larkana, Sindh, Pakistan; 2 Zahid Ali Shaikh, FCPS. Chandka Medical College Hospital, Shaheed Mohtarma Benazir Bhutto Medical University (SMBBMU), Larkana, Sindh, Pakistan; 3 Aftab Hussain Shah, FCPS, FRCP. Chandka Medical College Hospital, Shaheed Mohtarma Benazir Bhutto Medical University (SMBBMU), Larkana, Sindh, Pakistan; 4 Aneel Kumar, FCPS. Chandka Medical College Hospital, Shaheed Mohtarma Benazir Bhutto Medical University (SMBBMU), Larkana, Sindh, Pakistan

**Keywords:** Cirrhosis, Proton Pump Inhibitor, Spontaneous Bacterial Peritonitis

## Abstract

**Objectives:**

The current study aimed to determine the Spontaneous Bacterial Peritonitis (SBP) risk due to increased use of Proton Pump Inhibitors (PPIs) among cirrhotic patients with ascites.

**Methods:**

This retrospective case-control study was conducted at Chandka Medical College & Hospital, Larkana from March 2013 to February 2014, involving 215 cirrhotic patients with ascites. Paracentesis was performed to distinguish cirrhotic patients with SBP and Polymorphonuclear Neutrophil (PMN) count ≥ 250 neutrophils/mm^3^ (cases) and non-SBP with PMN count < 250 neutrophils/mm^3^ (controls). The demographic details, history of PPIs use before admission and duration of Chronic Liver Disease (CLD) were inquired and statistical analysis was carried through SPSS Version 23.0.

**Results:**

Increased pre-hospital PPI intake was observed among cirrhotic patients with SBP (69.8%) as compared to those without SBP (48.8%; p = 0.014). The mean duration of PPI use was 19.16 ± 4.772 days, and it was more significant among older cirrhotic patients (p < 0.05). Increased duration of CLD was observed among PPI users, i.e. 20.47 ± 6.305 months vs. 18.95 ± 5.527 months among non-PPI users (p < 0.05).

**Conclusions:**

Our results show that cirrhotic patients with ascites consuming PPIs are more likely to develop SBP as compared to non-PPI users.

## INTRODUCTION

Cirrhosis is a well-known late-stage liver disease characterized by inflammation, cell scarring and death, increasing the risk of bacterial infections. Hence it is associated with increased morbidity and mortality rate.^1^ There is a four-fold increase in the complexity and mortality rate with bacterial infections among cirrhotic patients,^2^ SBP has been documented as the most common and serious deadly complication observed among the cirrhotic patients with ascites.^3^ The pathogenetic pathway of SBP indicates that it is triggered by the translocation of the bacteria from the gut flora to mesenteric lymph nodes, facilitated by the increased permeability of the gut and the overgrowth of the intestinal bacteria.^3^

Infection susceptibility increases among cirrhotic patients due to impaired activity of the reticuloendothelial phagocytic system, complement deficiency and dysfunctional neutrophils.^4^ Additionally, gastric acid also provides a defensive activity against microorganisms. The proliferation of bacteria in the gut is one of the prime outputs of gastric acid reduction, predisposing an individual to bacterial infections.^5^ Majority of the patients encountering gastric acid-related disorders administer PPIs, i.e., 46 to 78% of the cirrhotic patients are involved in extensive use of PPIs.^6^ It has been effective in controlling acid associated problems, but its overuse is well-documented among cirrhotic patients,^7^ precipitating the enteric infection risk.^8^

As stated earlier, the cirrhotic patients are more vulnerable to enteric infections, besides this existing data also indicates the significant association of PPI use and SBP development among patients with cirrhosis and ascites, but the data pertinence is still debatable.^9^ PPIs therapy is known to alter the microbial flora in the small intestine, neutrophilic impairment, and delays gastric emptying.^8^ Moreover, decreased PPI metabolism also increases the infectious risk among cirrhosis patients, ultimately resulting in high PPI exposure.^10^

Although an increased risk of SBP has been recognized among PPI users, but contradictory literature also does exist. Therefore, the current study aimed to determine the association of PPI use with the development of SBP among cirrhotic patients with ascites in Chandka Medical College & Hospital, Larkana.

## METHODS

A case-control study was conducted at the Medicine Department of Chandka Medical College & Hospital, Larkana, for a duration of one year from March 2013 to February 2014. A total of 215 cirrhosis patients with ascites aged between 20 to 60 years were included in the study after obtaining written informed consent. After enrolment, each case was observed individually for the co-occurrence of SBP, the patients with PMN count ≥ 250 neutrophils/mm^3^ were SBP cases. In contrast, the non-SBP patients (controls) were cirrhotic patients with PMN count <250 neutrophils/mm^3^. Patients with the non-cirrhotic cause of ascites, secondary bacterial peritonitis, peritoneal tuberculosis or peritoneal carcinomatosis and those on chronic antibiotic prophylaxis or with antecedent gastrointestinal bleeding were excluded from the study.

Patient’s demographic details, history of PPIs use before admission and duration of CLD were inquired and entered into a pre-designed Performa. The PPI users and non-users were identified through interviews, and those consuming PPIs daily for the last two weeks were considered as PPI users. The collected data was analyzed using SPSS Version 23.0, categorical variables like gender and pre-hospital PPI intake were presented as frequency & percentages while the continuous variables like age and CLD duration were given as mean ± standard deviations (SD). Chi-square test was used to compare the rate of pre-hospital PPIs intake between cases and the controls, and p-value < 0.05 was considered significant. Stratification was done based on the gender, age and duration of CLD to observe the effect of the following on outcomes.

The Ethics Committee approved (Ref: SMBBMU/ORC/02, Dated: 24-06-2020) the study protocol of Shaheed Mohtrama Benazir Bhutto Medical University, and ethical conduct as per the standard guidelines for human-based research was ensured.

## RESULTS

A total of 215 cirrhotic patients with ascites were enrolled in the study, and SBP was confirmed among 43 patients (cases) while 172 non-SBP patients were placed in the control group, as shown in Table-I. Of the total, 140(65.1%) were males (30 cases & 110 controls) and 75(34.9%) were females (13 cases & 62 controls). The mean age of the study population was 47.85 ± 7.2 years, and the mean CLD duration was 19.8 ± 6.0 months. 114(53%) patients had a history of pre-hospital PPI intake with a mean duration of 19.16 ± 4.772 days.

**Table-I:** Demographic & Clinical Characteristics of the study population.

Characteristics	(n=215)
Mean age (Years)	47.85±7.2
***Gender***	
Male	140(65.1)
Female	75(34.9)
Mean duration of CLD (Months)	19.8±6.0
Pre-hospital PPIs intake	114(53)
Mean duration of PPIs intake (Days)	19.16±4.772
***SBP***	
Positive (Case)	43(20)
Negative (Control)	172(80)

*Spontaneous bacterial peritonitis (SBP); Proton pump inhibitors (PPIs); Chronic Liver Disease (CLD)

Out of the 43 cirrhotic patients positive for SBP, 30(69.8%) were involved in pre-hospital PPI intake, and 13(30.2%) were non-PPIs users. While among 172 cirrhotic patients negative for SBP, 84(48.8%) were PPI users, and 88(51.2%) were non-PPIs users. This difference of pre-hospital PPI use among the cases and controls was statistically significant (p = 0.014) (Table-II).

**Table-II:** Association of proton-pump inhibitors intake with spontaneous bacterial peritonitis.

	PPI users (n=114)	Non-PPI users (n=101)	P-value
Case (n=43)	30(69.8)	13(30.2)	0.014
Control (n=172)	84(48.8)	88(51.2)	

*Case-SBP Positive; Control-SBP Negative

The stratification of demographic and clinical characteristics of the PPIs user and non-users is given in Table-III. It was observed that 64.3% of males had a history of pre-hospital PPI intake as compared to 32% of females and the association was statistically significant (p < 0.05). The mean CLD duration was increased among the PPI users, i.e., 20.47 ± 6.305 months vs. 18.95 ± 5.527 months among the non-users (p < 0.05) (Table-III).

**Table-III:** Characteristics of the patients classified as per the use and non-use of proton pump inhibitors.

Characteristics	PPI users (n=114)	Non-PPI users (n=101)	p-value
***Gender***			
Male	90(64.3)	50(35.7)	<0.05
Female	24(32.0)	51(68.0)	
Age (Years)	48.46±7.299	47.17±7.006	<0.05
Duration of CLD (Months)	20.47±6.305	18.95±5.527	<0.05

*Values are given as n(%) or mean ± SD, *Proton pump inhibitors (PPIs); Chronic Liver Disease (CLD)

## DISCUSSION

The current findings support the existing literature regarding the PPIs intake among the cirrhotic patients, and increased risk of enteric infections, favoring the association of the PPIs use with the SBP development. It was found that 69.8% of the cirrhotic patients with positive SBP (case) were taking PPIs before hospital admission, and 30.2% were non-PPIs users, a significant association was found between the two variables (p < 0.05). Although the adverse effects of PPI use are evident but it has been extensively used, overused and even prescribed for years.^11^-^15^ The relationship between the PPI use and the associated side-effects has been underestimated and taken casually. The risk of gastric neoplasia, dementia, osteoporosis-related fractures, kidney and liver diseases is increased among the long-term PPI users.^16^ Moreover, the reactive oxygen intermediates were also impaired by human neutrophils in response to omeprazole administration. Hence, indicating the bactericidal activity due to reduced neutrophils.^17^

Our findings were also supported by another study, indicating a high occurrence of SBP among cirrhotic patients consuming PPIs.^18^ In contrast, the data from a retrospective study indicated no significant difference in the mortality rate among the cirrhosis patients using PPI (84%) or not using PPI (73%) (p = 0.30).^12^ While a Taiwanese case-control study confirmed the development of SBP among 947 patients out of 86,418 patients with advanced liver cirrhosis using acid suppression, i.e. higher risk of infection was associated with cumulative days of gastric acid suppression (p < 0.0001).^19^ PPIs were termed as a significant predictor of 30-day mortality among the cirrhosis patients with SBP by a parallel retrospective study, but these cases were observed with gastrointestinal bleeding and hepatocellular carcinoma, which might be the reason behind mortality other than the PPIs use.^20^ More recently, a cohort study from Brazil showed no difference in the rate of SBP development among PPI users and non-users.^21^ Though this association between PPI use and SBP development remains controversial and is being studied extensively, several pieces of evidence claim a direct correlation between the two. Confirming this relationship three meta-analyses, in 2011 reviewing four studies (n = 722; OR 2.77; 95% CI 1.82–4.23),^18^ in 2013 involving eight studies (n = 3,815; OR 3.15; 95% CI 2.09–4.74)^11^ and the third one conducted in 2015 including 17 studies (n = 8,204; OR 2.17; 95% CI 1.46–3.23).^22^ It was concluded that the PPI use among cirrhosis patients with ascites increases the SBP development risk to three folds, and there is a significant relationship between PPI use and development of SBP.^23^

The long-term use of PPI has been associated with enteric and respiratory infections, specifically among older cirrhotic patients with renal or liver comorbidities.^24^ The mean duration of pre-hospital PPI intake was 19.16 ± 4.772 days, although the hazard ratio (HR) is not estimated in our study but it is evident that the long-term PPI use increases the HR, i.e. for ≥ 1 year, the reported HR was 5.04, 6.65 (≥ 2 years) and 8.34 (≥ 3 years).^24^ The comparison of clinical and demographic characteristics of the PPI users and non-users is summarized in Table-III . It was observed that age was a significant predictor (p < 0.05), and PPI users were comparatively older as compared to non-PPI users, i.e., 48.46 ± 7.299 years vs. 47.17 ± 7.006 years. Elzouki et al., also presented similar results, age was significantly associated with PPI use (p = 0.0001).^15^ Besides, a contradictory study from Taiwan quoted no significant association between the demographic characteristics and PPI use, i.e., mean age of PPI users was 60.9 ± 13.8 years and 60.9 ± 13.6 years among non-PPI users (p = 0.952). ^25^ However, the effect of older age on the development of SBP requires further research as age may or may not be an independent risk factor in addition to PPI use.

### Limitations of the study

The severity and mortality due to PPI use and SBP were not evaluated, no laboratory tests were performed, and none of the data was included. Lastly, the duration of the PPI exposure is evaluated, but the HR associated with it isn’t documented. Hence, the study provided useful data indicating the association of PPI use and SBP development.

## CONCLUSION

The cirrhotic patients with ascites involved in pre-hospital PPI intake show greater susceptibility toward SBP development as compared to non-PPI users. However, our findings reinforce the positive correlation of PPI use and SBP as demonstrated by other retrospective case-control studies. Further prospective studies are required to evaluate the reduction in SBP development with restricted PPI use.

### Authors’ Contribution:

**BAS, ZAS, AHS, AK** conceptualized and designed the study.

**BAS, ZAS, AHS, AK** are responsible for literature review, data collection, analysis, manuscript writing and critical review.

**BAS, ZAS, AHS, AK** are equally responsible and accountable for the accuracy and integrity of the work.
